# Supplementary material for: Identification of TIAM1 as a Potential Synthetic-Lethal-like Gene in a Defined Subset of Hepatocellular Carcinoma
Source: Int J Mol Sci. 2023 Mar 28;24(7):6387. doi: 10.3390/ijms24076387 (PMC10094703; doi:10.3390/ijms24076387)
Supplement: Supplementary file 1 [file ijms-24-06387-s001.zip › ijms-2221645-supplementary.pdf]

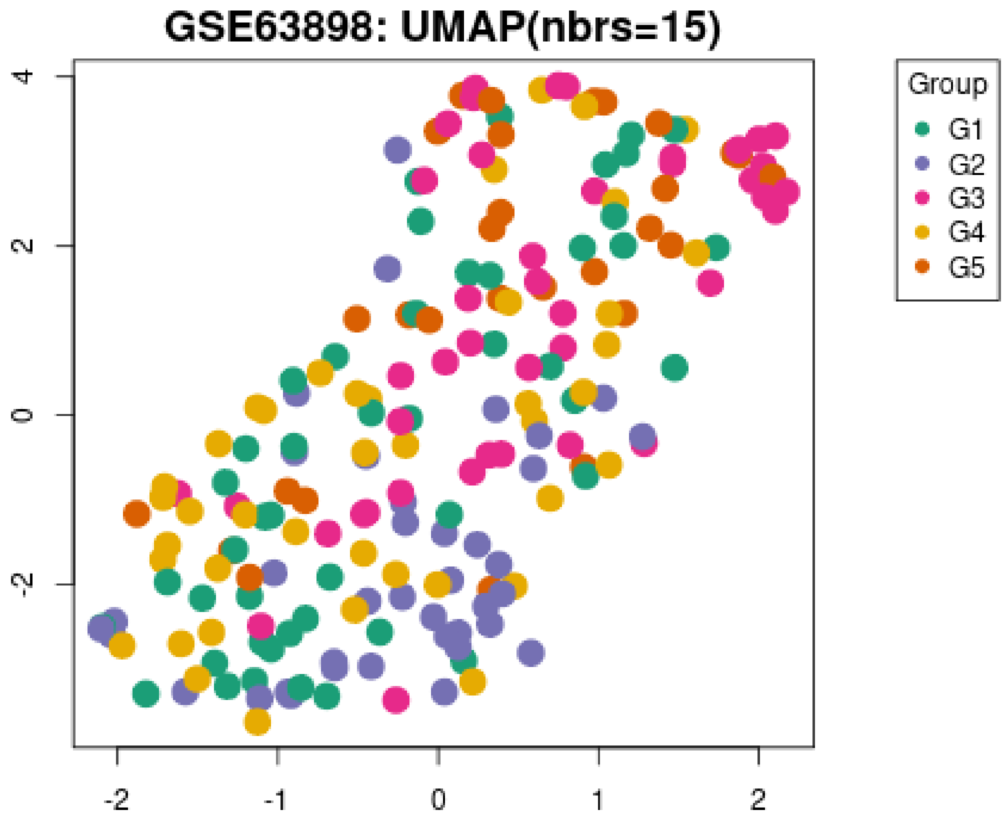


**Supplementary Figure S1: Clustering of HCC samples based on whole transcriptome.** HCC samples grouped from G1-G5 were analysed for UMAP (Uniform Manifold Approximation and Projection) by GEO2R online tool to visualize any patterns of clustering in the groups based on whole transcriptome data.

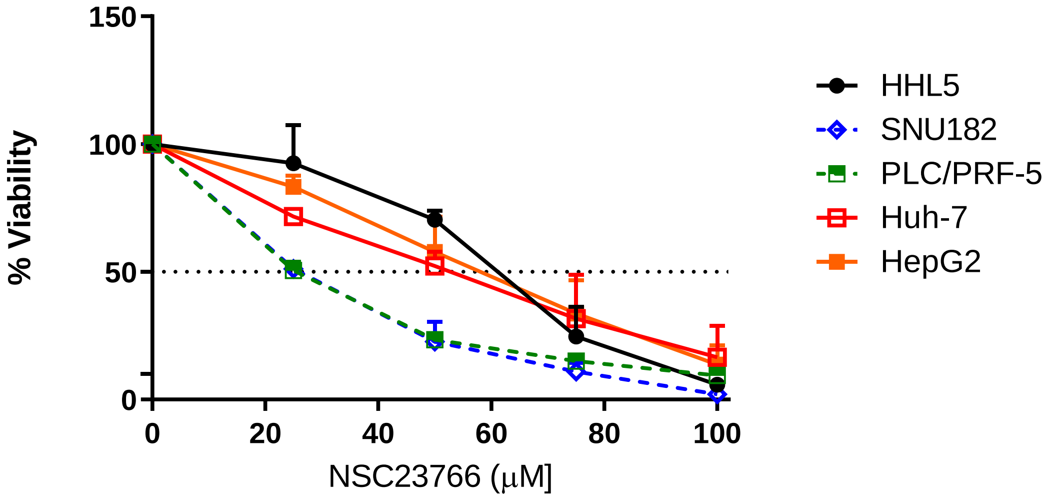


**Supplementary Figure S2: Growth inhibition curve for indicated cell lines upon treatment with NSC23766.** Cells were treated with NSC283766 and growth was determined by MTT assay, 6 days after treatment. Graph represents mean ± SD of 3 independent repeats done as 4 technical replicates.

**A**

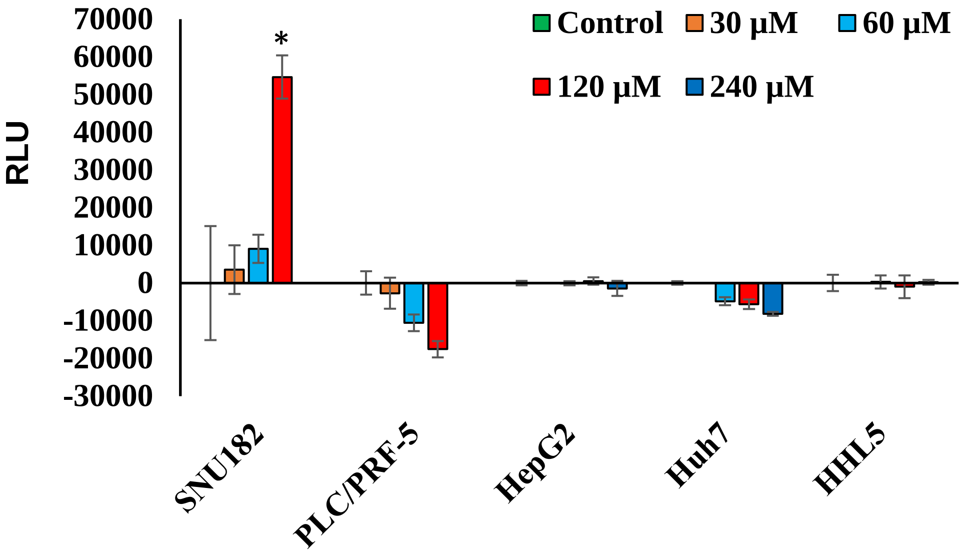


**B**

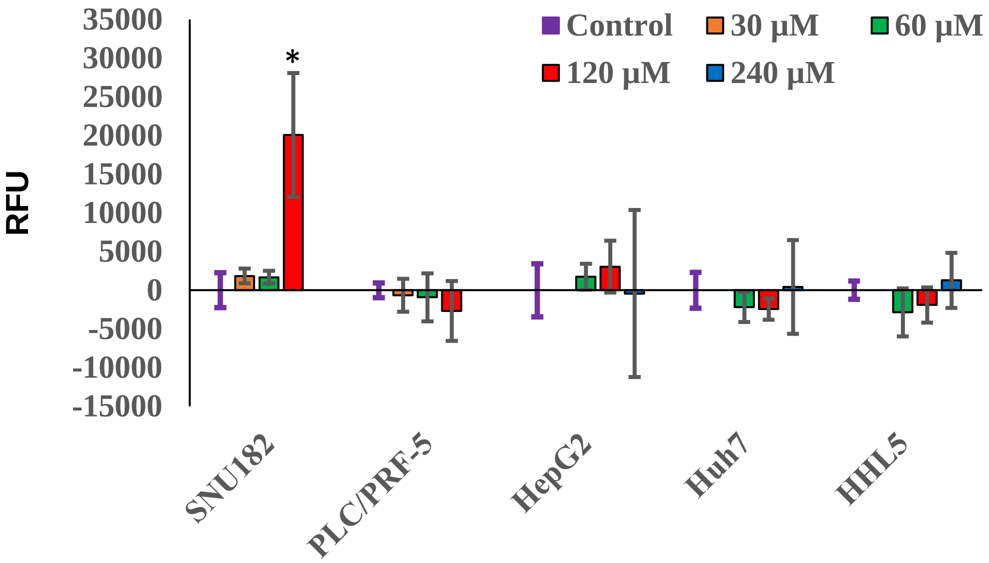


**Supplementary Figure S3: Cytotoxicity of NSC23766 on indicated cell lines.** (A) Apoptosis induction on indicated cell lines was evaluated by Caspase-Glo® 3/7 apoptosis assay (Promega) 48 hr after NSC23766 treatment at the indicated doses. After 48 hr of drug addition, apoptosis was assessed by measuring the luminescence signal 1hr after adding Caspase-Glo® 3/7 reagent as per the manufacturer's instructions. Graph represents relative luminescence unit (RLU) obtained by subtracting the control values for respective cell lines. Data is mean ± SD of two independent repeats done as 4 technical replicates. * $P < 0.00005$, t-test two-sample assuming equal variances. (B) Cell toxicity on indicated cell lines was evaluated by CellTox® assay (Promega). Cell toxicity was determined 48 hr after NSC23766 treatment at the indicated doses by measuring the fluorescence signal as per the manufacturer's instructions. Graph represents relative fluorescence unit (RFU) obtained by subtracting the control values for respective cell lines. Data is mean ± SD of two independent repeats done as 4 technical replicates. * $P < 0.005$, t-test two-sample assuming equal variances.

**Supplemental Table S1. Methylation levels at CpG sites in the differentially methylated regions in SSV gene candidates**

## TIAM1

| | | Cell lines | | | | |
|---|---|---|---|---|---|---|
| CpG ID | Gene | HepG2 | HUH-7 | HHL5 | PLC5 | SNU182 |
| cg16711792 | TIAM1 | 0.28 | 0.87 | 0.06 | 0.12 | 0.51 |
| cg01134185 | TIAM1 | 0.35 | 0.24 | 0.07 | 0.04 | 0.20 |
| cg00615473 | TIAM1 | 0.67 | 0.97 | 0.01 | 0.02 | 0.02 |
| cg02367930 | TIAM1 | 0.31 | 0.74 | 0.03 | 0.03 | 0.04 |
| cg13486354 | TIAM1 | 0.33 | 0.40 | 0.07 | 0.06 | 0.06 |
| cg27118059 | TIAM1 | 0.97 | 0.98 | 0.01 | 0.01 | 0.01 |
| cg06464111 | TIAM1 | 0.27 | 0.47 | 0.05 | 0.05 | 0.04 |
| cg12258307 | TIAM1 | 0.84 | 0.94 | 0.03 | 0.03 | 0.03 |
| cg25046584 | TIAM1 | 0.22 | 0.67 | 0.16 | 0.13 | 0.21 |
| cg16867777 | TIAM1 | 0.91 | 0.89 | 0.53 | 0.07 | 0.58 |
| cg15853125 | TIAM1 | 0.60 | 0.81 | 0.59 | 0.31 | 0.94 |
| | | Non-group2 | | Normal | Group2 | |
| | | 0.62 | | 0.15 | 0.16 | |

## LDHB

| | | Cell lines | | | | |
|---|---|---|---|---|---|---|
| CpG ID | Gene | HepG2 | HUH-7 | HHL5 | PLC5 | SNU182 |
| cg10195295 | LDHB | 0.89 | 0.93 | 0.07 | 0.54 | 0.51 |
| cg04949489 | LDHB | 0.12 | 0.71 | 0.02 | 0.09 | 0.12 |
| cg09130035 | LDHB | 0.95 | 0.96 | 0.03 | 0.50 | 0.38 |
| cg07869763 | LDHB | 0.20 | 0.85 | 0.03 | 0.04 | 0.06 |
| cg10483825 | LDHB | 0.87 | 0.93 | 0.04 | 0.66 | 0.58 |
| cg06437004 | LDHB | 0.63 | 0.71 | 0.04 | 0.42 | 0.42 |
| cg00753478 | LDHB | 0.97 | 0.98 | 0.02 | 0.59 | 0.42 |
| cg00501628 | LDHB | 0.93 | 0.95 | 0.04 | 0.55 | 0.72 |
| cg02659794 | LDHB | 0.97 | 0.98 | 0.03 | 0.80 | 0.45 |
| cg09274094 | LDHB | 0.99 | 0.99 | 0.02 | 0.90 | 0.70 |
| cg04354474 | LDHB | 0.25 | 0.84 | 0.05 | 0.27 | 0.17 |
| cg14209005 | LDHB | 0.82 | 0.97 | 0.03 | 0.34 | 0.24 |
| cg05578357 | LDHB | 0.57 | 0.96 | 0.02 | 0.15 | 0.03 |
| cg08148261 | LDHB | 0.94 | 0.96 | 0.06 | 0.93 | 0.62 |
| cg20717612 | LDHB | 0.84 | 0.86 | 0.08 | 0.61 | 0.42 |
| cg03243946 | LDHB | 0.56 | 0.87 | 0.04 | 0.29 | 0.24 |
| | | Non-group2 | | Normal | Group2 | |
| | | 0.81 | | 0.04 | 0.43 | |

**Table S2: Details of RT-qPCR experimentation according to MIQUE guidelines.**

| | |
|---|---|
| Definition of experimental and control groups | Described in manuscript and figure legends |
| number within each group | Described in manuscript and figure legends |
| Description | Described within manuscript |
| Processing procedure | As detailed in methods |
| How sample was frozen | Cells were snap frozen |
| Samples storage and duration | Stored in -80C for varying lengths of time |
| **Nucleic acid extraction** | |
| Procedure | Total RNA purification kit (Norgenbiotek) |
| Contamination assessment | Nanodrop |
| nucelic acid quantification | Nanodrop |
| RNA integrity | Not done as RNA is used for RT-qPCR application only |
| Inhibition testing | The standard curve to assess primer effeciency was also used to rule out the presence of any inhibitors |
| **Reverse transcription** | |
| Complete reaction conditions | High-Capacity cDNA Reverse Transcription kit (Applied biosystems) as per manufacturer's instructions |
| Amount of RNA | 1ug in 20 ul reaction vol |
| priming oligonucleotide | Random primers |
| reverse transcriptase and concentration | As per the kit |
| temperature and time | As per the kit |
| **qPCR target info** | |
| Gene symbol | Included in methods section |
| sequence accession number | Included in methods section |
| Amplicon length | Included in methods section |
| in silico specificity screen | Completed for each target gene when designing primers using NCBI primer blast |
| Location of each primer by exon intron | Primer pair were separated by at least one intron on the corresponding genomic DNA in all the cases |
| **qPCR oligonucleotides** | |
| Primer sequences | Included in methods section |
| loation and identity of any modifications | NA |
| **qPCR protocol** | |
| complete reaction conditions | See methods |
| reaction vol | 10ul |
| amount of cDNA | 2ul |
| primer, probe and dNTP concentrations | See methods |
| Polymerase identity | See methods |
| buffer / kit identity and manufacturer | See methods |
| Additives (SYBR, DMSO etc) | SYBR |
| Complete thermocycler parameters | Applied Biosystems QS7 machine, manufacturer recommended settings for Thermo fisher Life technologies SYBR mix, exactly as stated |
| Manufacturer of qPCR instrument | Applied Biosystems QS7 |

| | |
|---|---|
| **qPCR validation** | |
| Specificity, gel, sequence, melt or digest | Melt curve analysis performed for each sample |
| Calibration curve with slope and y intercept | For all primers used we have completed a calibration curve and calculated the efficiency of the primer pair |
| PCR efficiency calculated from slope | See above |
| r2 of calibraion curve | Calculated for each primer pair |
| linear dynamic range | Curves were calculated from 3-4 dilutions in order to determine range |
| Cq variation at LOD | NA |
| Evidence for LOD | Instead of determning LOD, we utilised primer standard curves to make sure that the samples Cq fall in the linear range |
| **Data analysis** | |
| qPCR analysis programme | QuantStudio |
| Method of Cq determination | See above |
| outlier identification and disposition | In excel, variation in Cq used to detemine outliers, these were discarded from analysis |
| results for NTCs | The signal of the amplification plot was mostly undetermined or very late (Cq>35) |
| Justification for number and choice of reference genes | HPRT1 used as Cq values for this was very similar between all samples analysed within an assay |
| descrition of normalisation method | Described in methods and/or figure legends |
| technical replicates | 3-4 technical replicates were run per sample on each qPCR plate |
| repeatability | All assays shown were performed multiple times on biological replicate samples and were repeatable |
| statistical methods | See methods and fig. Legends for details |
| software | Microsoft Excel and Graphpad prism |
